# Toward Improving the Transition of Patients With Congenital Adrenal Hyperplasia From Pediatrics to Adult Healthcare in Japan

**DOI:** 10.3389/fped.2022.936944

**Published:** 2022-06-21

**Authors:** Kei Takasawa, Kenichi Kashimada

**Affiliations:** Department of Pediatrics and Developmental Biology, Tokyo Medical and Dental University (TMDU), Tokyo, Japan

**Keywords:** healthcare transition, congenital adrenal hyperplasia, 21-hydroxylase deficiency, childhood-onset endocrine disease, inter-professional support system

## Abstract

The transition of patients with childhood-onset chronic diseases from pediatric to adult healthcare systems has recently received significant attention. Since 2013, the Japan Pediatric Society developed working groups to formulate guidelines for transition of patients with childhood-onset chronic diseases from pediatric to their disease specialty. Herein, we report on the activities of the Japan Society of Pediatric Endocrinology (JSPE) and the current status of transition medicine for 21-hydroxylase deficiency (21-OHD) in Japan. The JSPE proposed roadmaps and checklists for transition and prepared surveys on the current status of healthcare transition for childhood-onset endocrine diseases. In Japan, newborn screening for 21-OHD started in January 1989; however, there is no nationwide registry-based longitudinal cohort study on 21-OHD from birth to adult. The current status and the whole picture of healthcare and health problems in adult patients with 21-OHD remain unclear. Thus, we conducted a questionnaire survey on JSPE members to clarify the current status of healthcare transition of 21-OHD and discuss future perspectives for the healthcare transition of patients with 21-OHD in Japan.

## Introduction

Advances in pediatrics and neonatal medicine have dramatically improved the prognosis of children with chronic diseases, which have consequently increased the number of adolescents transitioning from pediatric to adult healthcare systems. The transition of patients with childhood-onset chronic diseases from pediatric to adult healthcare systems has recently received significant attention worldwide (1). The transition has been defined as a multifaceted, active process that attends to the medical, psychosocial, and educational needs of adolescents as they move from pediatric to adult healthcare systems (2). The number of adolescents undergoing healthcare transition is increasing; therefore, suitable and individually optimized programs are required to integrate the patients into adult-centered care and to help them grow socially and become independent working adults (1).

In Japan, the term “healthcare transition” was introduced in 2006 to replace “carryover,” and the Japan Pediatric Society convened a committee for healthcare transition and summarized its statements in 2013 (3). Around the same time, a Research Committee on the Investigation and Refined Policy to Support Social, Medical, and Educational Life of Children with Chronic Disease was appointed by the Ministry of Health, Labour and Welfare of Japan to develop a support guidebook on healthcare transition for pediatricians (4). In response to the statement, subcommittees of the Japan Pediatric Society created working groups for transition and started to formulate guidelines for transition into their disease specialty. In the present review, we report the activities of the transition committee of the Japan Society of Pediatric Endocrinology (JSPE) and the current status of transition medicine for childhood-onset endocrine diseases in Japan, taking 21-hydroxylase deficiency (21-OHD) as an example.

## Healthcare Transition of Patients With Childhood-Onset Endocrine Diseases in Japan

Most childhood-onset endocrine diseases cannot be cured and need lifelong treatment. The statement for healthcare transition of the JSPE advocated that healthcare “transition” does not necessarily equate to “transfer” from pediatrics to adult healthcare (5). Healthcare transition is a lifelong process that should be started at diagnosis and includes education or support suited to each patient’s development stage. The transition committee of the JSPE proposed roadmaps and checklists of transition aiming for and supporting self-reliance and autonomy of patients with childhood-onset endocrine diseases, including 21-OHD, type 1 diabetes mellitus (T1DM), combined pituitary hormone deficiency, and Prader-Willi syndrome (6). The transition from pediatric to adult healthcare should be based on transitioning the initiative for treatment from parents to patients themselves with individualized, planned, organized, and multidisciplinary support. Moreover, support systems should be constructed with interprofessional work inside and outside facilities and should be done in cooperation with each community.

Although the significance and necessity of healthcare transition have been widely recognized, the actual condition of healthcare transition of patients with childhood-onset endocrine diseases in Japan remains unclear. Although a person to supervise the transfer of a T1DM patient, i.e., a diabetologist, may be relatively easy to find and access compared with finding someone to supervise the transfer of patients with other congenital or childhood-onset endocrine diseases, there still are no systems for healthcare transition that have been established. A questionnaire survey of pediatric endocrinologists on the current status of healthcare transition for patients with T1DM in Japan revealed that 61.9% of pediatric endocrinologists continue to treat adult patients with T1DM mainly because of the patients’ own request (7). Another cohort study indicated that one-fourth of patients over 40 years of age with childhood-onset T1DM received pediatric care (8). Surveys on the current status of healthcare transition of other rarer childhood-onset endocrine diseases are needed, and some of them are being prepared by the JSPE.

## Healthcare Transition of Patients With 21-Hydroxylase Deficiency in Japan

The most common form of congenital adrenal hyperplasia (CAH), which is a group of autosomal recessive disorders characterized by cortisol synthesis deficiency, is 21-OHD. Because patients with classical 21-OHD require lifelong steroid replacement, it requires management for each stage of life. Undertreatment leads to adrenal insufficiency and hyperandrogenism, reducing adult height because of premature induction of puberty, while overtreatment leads to obesity and Cushing syndrome and inhibits growth (9). Therefore, it is crucial to control the treatment. Practical administration should be individually dependent on the patient’s condition and age (9). During the pediatric period, the main targets for treatment are normal physical growth, normal sexual development, and avoidance of an adrenal crisis. Once growth is completed, a shift in treatment goals from optimal growth and puberty to the prevention of long-term adverse outcomes and optimization of sexual function and fertility is needed. The clinical practice guidelines in the United States and Europe focused on the significance of healthcare transition in the long-term management of patients with CAH, recommended the gradual transition of adolescents to adult care over several years, and suggested the use of joint clinics comprised of pediatric, reproductive, and adult endocrinologists during this transition (10). Indeed, the successful transition to adult endocrinologists from pediatricians is associated with regular medical follow-up and better health-related quality of life in adult patients with CAH (11).

In addition to the classical problems (adrenal failure and androgen excess), recent studies have shown that, in both adolescents and adults with CAH, morbidity is increased and quality of life is decreased by a number of causes, including obesity, hypertension, diabetes mellitus, impaired glucose tolerance, dyslipidemia, osteoporosis, and infertility (11–16). A large cohort study in the United Kingdom revealed a higher hazard ratio for all-cause mortality of 5.17 and a lower mean age at death (54.8 years) in patients with CAH than in the healthy control group (17). Health problems of patients with 21-OHD due to overtreatment or undertreatment, including metabolic and cardiovascular issues, fertility in both women and men, gonadal and adrenal tumors, and bone problems, may have subclinically developed in childhood or adolescence and should be managed in childhood from the aspect of adult healthcare (18); this requires detailed longitudinal patient data that are supported by seamless transition care from childhood to adulthood. However, recent cohort studies on patients with CAH in Europe revealed that many adult patients with CAH (10–50% depending on the study) did not receive follow-up care with an endocrine specialist after transition (19, 20).

The condition is similar in Japan, where newborn screening for 21-OHD started in January 1989 and one per 18,000 to 20,000 infants is found to have 21-OHD (18, 21, 22). Newborn screening promotes early recognition and treatment of infants with classic 21-OHD, consequently reducing morbidity and mortality (10, 23). However, there is no nationwide registry-based longitudinal cohort study on 21-OHD from birth to adulthood. A recent questionnaire survey on JSPE members showed that at least 10% of adult patients with classic 21-OHD after starting the newborn screening were treated by pediatric endocrinologists in Japan (24); however, longitudinal surveys and follow-up on the other 90% who transferred to adult healthcare may be required. The current status and the whole picture of healthcare and health problems in adult patients with 21-OHD remain unclear.

To better understand the current status of the follow-up systems for patients with 21-OHD, we sent cross-sectional questionnaire surveys to all 190 councilors of the JSPE (24). The study consisted of two parts including an opinion survey of pediatric endocrinologists treating adult patients with 21-OHD on healthcare transition and a fact-finding survey on adult patients with 21-OHD who were treated in pediatrics. In the first part of the study, the biggest factor hindering the healthcare transition for 21-OHD (selected by 63% of pediatric endocrinologists who took the survey) was insufficient knowledge and experience of physicians involved in adult healthcare for 21-OHD. The second part of the study focused on 115 patients (53 men and 62 women with a median age of 26). It revealed that half of them continued to be treated in pediatrics at their own request. The prevalence of long-term complications including obesity, osteoporosis, infertility, menstrual disorder, gender dysphoria, and testicular adrenal rest tumor was 27.5, 8.8, 11.1, 26.3, 7.1, and 12.5%, respectively, which is comparable to previous reports (10, 14, 25–30). However, more than half of the patients were not assessed for the presence or absence of osteoporosis and infertility. Although 44 of the 62 female patients had genital reconstructive surgery, more than half of them were not followed up by gynecologists or pediatric urologists in parallel with pediatric endocrinologists.

The major points obtained from the survey are as follows:

(1) Pediatric endocrinologists who treated adult patients with 21-OHD regarded the problems of counterparts as hindrances to healthcare transition; however, the major reason for continuing to be treated in pediatrics was the patient’s request.
(2) One-fourth of the adult patients exhibited obesity, and many of them developed obesity in their 20s.
(3) More than half of the adult patients with 21-OHD treated in pediatrics were not checked for infertility and osteoporosis.

**FIGURE 1:**
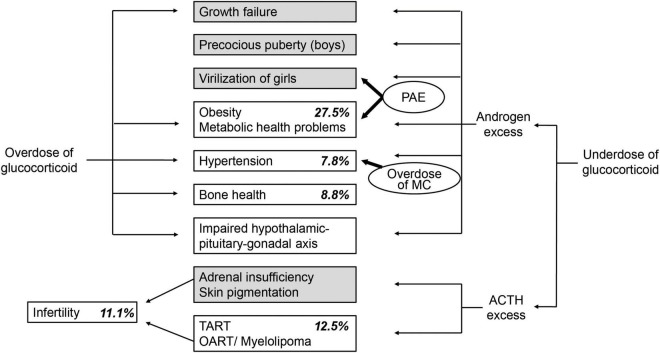
Endocrine imbalances in 21-hydroxylase deficiency (21-OHD) (18) and long-term complications of adult patients with 21-OHD treated in pediatrics in Japan (24). *Italic* indicates the prevalence of long-term complications of adult patients with 21-OHD treated in pediatrics in Japan. Gray boxes indicate complications that mainly draw the attention of pediatric endocrinologists. PAE, prenatal androgen excess; MC, mineralocorticoid; ACTH, adrenocorticotropic hormone; TART, testicular adrenal rest tumor; OART, ovarian adrenal rest tumor.

The first finding indicates that there is a gap in perception of healthcare transition between the pediatric endocrinologists and the adult patients with 21-OHD treated in pediatrics and suggeststhe necessity of education and psychological support for self-reliance and autonomy of the patients. The second finding suggests that there is a need for tightening the control of treatments to prevent childhood obesity in order to achieve a smooth transition to adult healthcare. The third finding suggests that there is a need not only for the enlightenment of pediatric endocrinologists but also for interprofessional and multidisciplinary cooperation with gynecologists, reproductive or adult endocrinologists, orthopedists, and liaison psychiatry teams. Health problems associated with adult patients with 21-OHD and the prevalence of the problems in the cohort are summarized in Figure 1 (18, 24).To prevent long-term complications including metabolic, gynecological, urological, orthopedic, and psychological problems and improve quality of life and social health, multifaceted and multidisciplinary support systems tailored to regional and individual characteristics are necessary (10, 11, 31). Medical staff of joint clinics with pediatric and adult healthcare have been suggested to optimize communication during the transition from pediatric to adult care (10), although these are not yet available for most patients worldwide (32). Pediatric endocrinologists need to take a more active approach to working with other healthcare workers, their patients, and society as a whole.

## Future Perspective of Healthcare Transition in 21-Hydroxylase Deficiency in Japan

We reviewed the current condition and issues to be addressed in the healthcare transition for childhood-onset endocrine diseases, especially 21-OHD, in Japan and indicated that there is plenty of room for improvement. Prior to, or in parallel with, building interprofessional support systems of health transition tailored to regional and individual characteristics, a more detailed grasp of the actual situations of health transition in 21-OHD from birth to adult is required. Because almost all patients with 21-OHD are diagnosed in the neonatal period by newborn screening, it should be possible to launch a nationwide disease registry and longitudinal follow-up system.

Multiple alternative treatment approaches are being developed with the aim of tailoring therapy for improved long-term outcomes for patients with 21-OHD, including treatments designed to replace cortisol in a physiological manner and treatments with adjunct agents intended to control excess levels of androgen, which thereby enables reduction in glucocorticoid doses, e.g., modified-release hydrocortisone, continuous subcutaneous hydrocortisone infusion pump, 17-hydroxylase inhibitor, hypothalamic–pituitary–adrenal axis suppressors, and cell-based or gene-based therapies (33). These advances in treatment may shed more light on the significance and necessity of healthcare transition.

The very first step toward the establishment of healthcare transition in 21-OHD may be to organize an interprofessional team for adolescent and young adult patients with 21-OHD in each facility or region at the initiative of pediatric endocrinologists while involving physicians or adult endocrinologists. The gradual transition of leadership from pediatric to adult endocrinologists will be fostered through participation and cooperation with activities of interprofessional support teams. Concurrently, it will be required to enlighten medical staff involved in adult healthcare and to promote patient education for health autonomy according to the stage of patient’s growth and development as a preparatory step for a gradual transition. Accumulation of interactive cooperation between interprofessional support teams tailored to the characteristic of each facility or region could form the foundation of healthcare transition in 21-OHD in Japan. The missions of pediatric endocrinologists in Japan are to disseminate the significance and necessity of healthcare transition in 21-OHD, attract participants across many different fields into support teams, and take on the role of a hub in interprofessional and multidisciplinary cooperations.

## Author Contributions

KT and KK designed and wrote the manuscript. KK supervised the drafting of the manuscript. Both authors contributed to the article and approved the submitted version.

## Conflict of Interest

The authors declare that the research was conducted in the absence of any commercial or financial relationships that could be construed as a potential conflict of interest.

## Publisher’s Note

All claims expressed in this article are solely those of the authors and do not necessarily represent those of their affiliated organizations, or those of the publisher, the editors and the reviewers. Any product that may be evaluated in this article, or claim that may be made by its manufacturer, is not guaranteed or endorsed by the publisher.
